# Machine learning models to identify low adherence to influenza vaccination among Korean adults with cardiovascular disease

**DOI:** 10.1186/s12872-021-01925-7

**Published:** 2021-03-09

**Authors:** Moojung Kim, Young Jae Kim, Sung Jin Park, Kwang Gi Kim, Pyung Chun Oh, Young Saing Kim, Eun Young Kim

**Affiliations:** 1 School of Medicine, Gachon University, Incheon, South Korea; 2 Department of Biomedical Engineering, Gachon University College of Medicine, Incheon, South Korea; 3 Department of Internal Medicine, Gil Medical Center, Gachon University College of Medicine, Incheon, South Korea; 4 Department of Radiology, Gil Medical Center, Gachon University College of Medicine, Incheon, South Korea

**Keywords:** Machine learning, Influenza vaccination, Cardiovascular disease

## Abstract

**Background:**

Annual influenza vaccination is an important public health measure to prevent influenza infections and is strongly recommended for cardiovascular disease (CVD) patients, especially in the current coronavirus disease 2019 (COVID-19) pandemic. The aim of this study is to develop a machine learning model to identify Korean adult CVD patients with low adherence to influenza vaccination

**Methods:**

Adults with CVD (n = 815) from a nationally representative dataset of the Fifth Korea National Health and Nutrition Examination Survey (KNHANES V) were analyzed. Among these adults, 500 (61.4%) had answered "yes" to whether they had received seasonal influenza vaccinations in the past 12 months. The classification process was performed using the logistic regression (LR), random forest (RF), support vector machine (SVM), and extreme gradient boosting (XGB) machine learning techniques. Because the Ministry of Health and Welfare in Korea offers free influenza immunization for the elderly, separate models were developed for the < 65 and ≥ 65 age groups.

**Results:**

The accuracy of machine learning models using 16 variables as predictors of low influenza vaccination adherence was compared; for the ≥ 65 age group, XGB (84.7%) and RF (84.7%) have the best accuracies, followed by LR (82.7%) and SVM (77.6%). For the < 65 age group, SVM has the best accuracy (68.4%), followed by RF (64.9%), LR (63.2%), and XGB (61.4%).

**Conclusions:**

The machine leaning models show comparable performance in classifying adult CVD patients with low adherence to influenza vaccination.

## Background

Influenza is an infectious disease of the respiratory system and the outbreak of influenza occurs worldwide in a seasonal manner, usually during the winter season in temperate climates. It is highly contagious and affects people via droplet contact. Influenza increases the morbidity and mortality among sufferers of cardiovascular disease (CVD), diabetes mellitus, asthma, obstructive pulmonary disease, and malignancy, who have higher chances of suffering from serious medical complications [1–3]. CVD is the leading global cause of death; approximately 17.9 million people died from CVD in 2016, accounting for 31% of all global deaths. Heart attack and stroke together contributed 85% of these deaths [4]. It is very important to recognize that CVD patients suffer from higher morbidity and mortality rates when they are infected with influenza. Several meta-analyses and systematic reviews have revealed a strong association between influenza infection and acute myocardial infarction [3, 5]. These reviews strongly recommend influenza vaccination for CVD patients.

The coronavirus disease 2019 (COVID-19) is caused by severe acute respiratory syndrome coronavirus 2 (SARS-CoV-2) and was declared a pandemic by the World Health Organization (WHO), on March 11, 2020 [6]. In the COVID-19 pandemic era, patients with comorbidities have an increased case fatality ratio, including 6.0% for hypertension, 7.3% for diabetes, and 10.5% for CVD [7]. With currently no proven vaccines or antiviral treatments, standard public health preventive efforts are being applied. To prevent the twindemic, where the COVID-19 pandemic and the 2020–21 influenza epidemic overlap, it is important to enhance influenza vaccination during the upcoming winter seasons.

In our previous study [8], we found that the vaccination coverage rate is low in non-elderly (< 65 years) CVD patients. To promote influenza vaccination coverage in CVD patients, it is necessary to identify a high risk population with low influenza vaccination adherence.

Machine learning algorithms build a model based on sample data, known as "training data", to make predictions or decisions without being explicitly programmed. As a subcategory of artificial intelligence, machine learning analyzes the patterns of the data and performs simultaneous tests on numerous variables to develop prediction models [9]. Therefore, this technique has a relative advantage over traditional statistical methods that can only address a small number of variables and cannot identify complex interactions between these variables.

The objective of this study was to create a model to predict influenza vaccination adherence using four different machine learning techniques: logistic regression (LR), support vector machine (SVM), random forest (RF), and extreme gradient boosting (XGB).

## Materials and methods

### Data collection and preparation

Data were obtained from the Fifth Korea National Health and Nutrition Examination Survey (KNHANES) conducted by the Korea Center for Disease Control and Prevention (KCDC) from January 2010 to December 2012. The dataset and questionnaire is provided with guidelines for calculating a health-related index through the KCDC online site (https://knhanes.cdc.go.kr/knhanes/eng/index.do).

KNHANES is a data resource operated by the KCDC since 1998. Researchers can access and use the data to support health-related studies. This nationally representative, cross-sectional survey includes approximately 10,000 participants every year and collects comprehensive data on their socioeconomic and nutritional status, lifestyle, physical activity, and health resource utilization through the three methods of health interviews, health examinations, and nutrition surveys. These three methods were conducted by professional staff members, including physicians and health interviewers. In the KNHANES, written informed consent was provided by every participant. The KNHANES survey was approved by the Institutional Review Board of the Korea Centers for Disease Control and Prevention (IRB No. 2010-02CON-21-C, 2011-02CON-06-C, 2012- 01EXP-01-2C). Further ethical approval for the use of KNHANES data are not required because publicly available datasets were used in this study.

From KNHANES V, 19,599 adults (> 19 years old) were selected by proportional allocation-systemic sampling with multistage stratification. Of the 19,599 participants in this sample, 17,872 participants have accessible information about their CVD and influenza vaccination status. Of these 17,872 participants, 815 had CVD; thus, 815 participants were included in the analysis (Table 1).

**Table 1:** Baseline characteristics of Korean adults with cardiovascular disease (CVD)

Parameters	Korean adults with CVD (%) (n = 815)
Sex, male	53.0 (2.2)
Age (mean, yr)	64.8 (0.5)
*Age group*	
19–49	7.6 (1.3)
50–64	39.3 (2.3)
≥ 65	53.1 (2.3)
Spouse, yes	77.5(1.8)
*Household income*	
Lowest quartile	40.1 (2.2)
2nd and 3rd quartile	44.3 (2.2)
Highest quartile	15.6 (1.7)
*Education*	
Less than elementary school	49.9 (2.1)
Middle or high school	39.3 (2.0)
College and above	10.8 (1.3)
*Insurance*	
National health insurance	93.6 (1.1)
Medical aid	6.4 (1.1)
Private Insurance, yes	40.3 (2.0)
Body mass index (mean, kg/m^2^)	24.5 (0.1)
Obesity (BMI ≥ 25 kg/ m^2^)	41.6 (2.2)
Alcohol drinker	14.8 (1.9)
Current smoker	18.9 (1.9)
Routine exerciser	43.1 (2.2)
Hypertension	62.2 (2.0)
Diabetes mellitus	25.8 (1.8)
Hyperlipidemia	32.7 (2.1)
Cancer	5.0 (0.8)
*Perceived health status*	
Good to very good	13.4 (1.4)
Normal	37.9 (2.1)
Poor to very poor	48.7 (2.1)
Health screening	61.0 (2.1)
Influenza vaccination	61.4 (2.2)

Abbreviations: BMI, body mass index

### CVD

Participants were considered to have CVD if they have been diagnosed by a physician based on the survey question of “if they had been ever diagnosed with CVD (any of the following conditions: stroke, myocardial infarction, and angina pectoralis) by a doctor”.

### Influenza vaccination

As the dependent variable, the influenza vaccination status was assessed using the question ‘‘have you received an influenza vaccination within the past 1 year?”, to which the participant answered ‘‘yes” or ‘‘No”.

### Independent variables

The independent variables included sociodemographic variables (sex, age, marital status, household income, education level, health insurance status, and private insurance), health-related lifestyle factors (height, weight, BMI, current smoking, drinking, exercise, and recent health screening), and health status (past history of hypertension, diabetes mellitus, hyperlipidemia, and cancer, and obesity and perceived health status).

The socio-demographic variables comprised the current age (19–49, 50–64, and ≥ 65 years), marital status (unmarried, separated, widowed, and divorced subjects were allocated a “no spouse” status), national insurance state (national health insurance, medical aid, or neither), and private health insurance state (yes, no). The household income level was classified into national quartile groups (lowest, 2nd, 3rd and highest quartile groups). The education background was divided into three categories, namely, less than elementary school, middle/high school, and college or above.

The health-related lifestyle factors included height, weight, and BMI, all of which are continuous variables. The BMI was calculated by dividing the weight (kg) by the square of the height (m^2^). The currently smoking variable has two categorical values, namely, yes and no. The recent health screening variable was set to "yes" for participants who replied that they had undergone health screening within the last two years. The exercise variable was set to “yes” for participants who walked or ran for at least 30 min more than 3 times a week.

The health status encompassed participants' past histories of hypertension, diabetes mellitus, hyperlipidemia, and cancer as categorical “yes” or “no” variables. Obesity was also a categorical variable and was divided into two groups: BMI above 25 kg/m^2^ and BMI below 25 kg/m^2^. Finally, the perceived health status was divided into three categories: good, normal, and bad.

### Models to predict influenza vaccination status

**Fig. 1:**
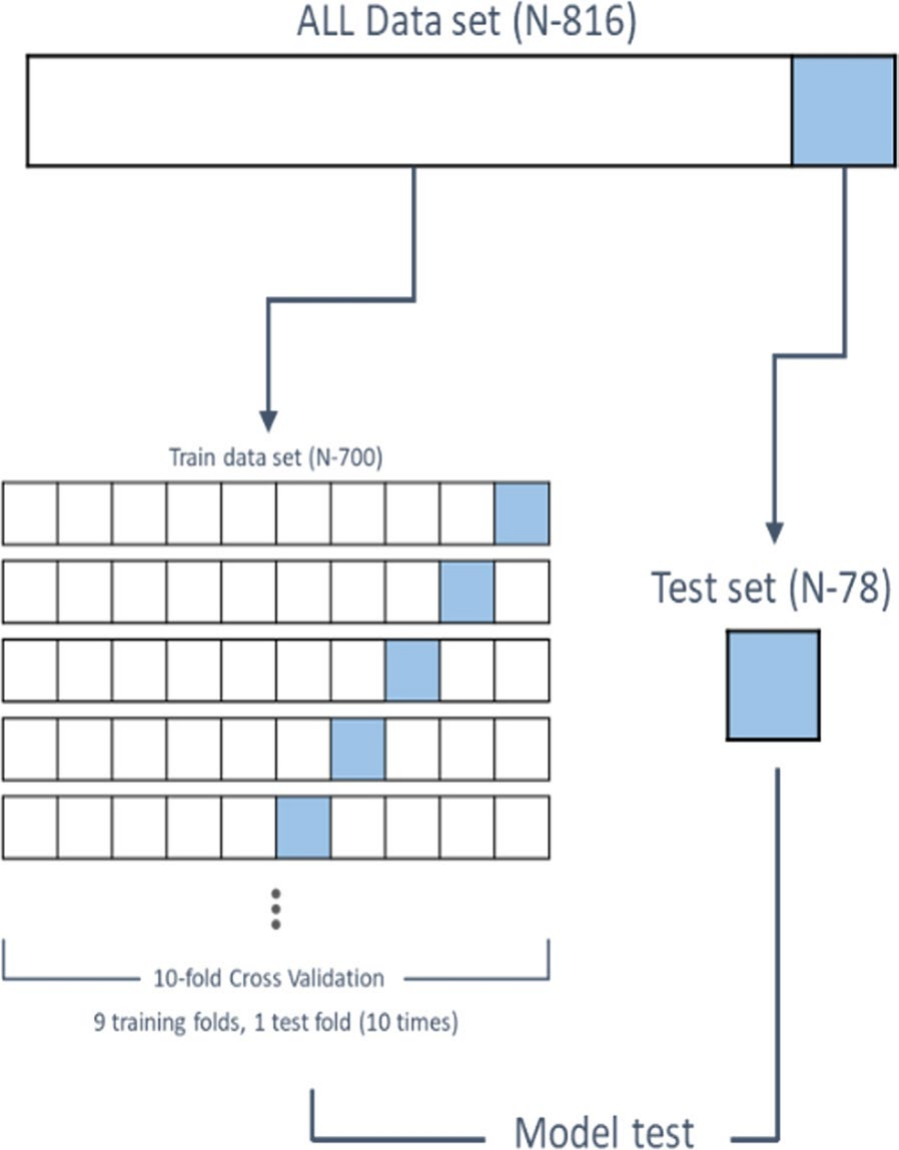
Prediction model development scheme

A prediction model for the influenza vaccination status was developed separately for two age groups: older than or equal to 65 years old, and younger than 65 years old. The alcohol consumption categorical variable was excluded from the model because there were missing data for this variable. Therefore, out of the 815 people with CVD (500 people with vaccination and 315 people without vaccination), 778 were ultimately selected, as shown in Table 2 and Fig. 1.

**Table 2:** Prediction model development after dividing dataset into two age groups

Korean adults with cardiovascular disease		Training data	Test data	Total
≥ 65 years	Influenza vaccination, no	70	8	78
	Influenza vaccination, yes	376	42	418
	Total	446	50	496
< 65 years	Influenza vaccination, no	157	17	174
	Influenza vaccination, yes	97	11	108
	Total	254	28	282

To prevent overfitting, a tenfold cross validation was used. The original sample dataset was randomly partitioned into 10 equal sized subsamples. Out of the 10 groups, 9 groups were used as the training sets for prediction models. The remaining group was used as the test dataset. Finally, Four models were developed with the training datasets using the LR, SVM, RF, and XGB machine learning techniques.

### Statistical analysis

The results are presented as percentages for the categorical variables and as means (± standard deviation) for the continuous variables. Logistic regression analysis was used to identify the factors associated with influenza vaccination adherence in the two age groups of above and below 65 years of age.

The machine learning models for classifying adherence to influenza vaccination were also developed separately for the two different age groups. A confusion matrix was used for measuring the diagnostic performance of each models in terms of the true positive (TP), false negative (FN), false positive (FP), and true negative (TN) rates, and the accuracy in predicting vaccination adherence among CVD patients. In addition, the area under the receiver operating characteristic curve (AUC) for each machine learning technique model was calculated to evaluate the general prediction performance on the test dataset.

The development of machine learning models and the analysis of the diagnostic performance were implemented using the open-source statistical software Python version 3.6.0. *P*-values of less than 0.05 (two-sided) were considered to be significant.

## Results

The univariable and multivariable logistic regression analysis results are summarized in Table 3 and Table 4. Among CVD patients aged ≥ 65 years, there are two significant variables associated with influenza vaccination, namely, the sex and national insurance state. Males (odds ratio [OR], 0.34 within a 95% confidence interval [95% CI], 0.14–0.84) and recipients of medical aid (OR, 0.34; 95% CI, 0.15–0.79) were less likely to receive influenza vaccination according to Table 3. In addition, recent health screening (OR, 1.97; 95% CI, 1.15–3.35) is associated with high adherence to influenza vaccination.

**Table 3:** Univariate and multivariate logistic regression analysis to identify factors associated with influenza vaccination status among Korean adults with cardiovascular disease (≥ 65 years)

	Crude OR	(95% CI)	Adjusted OR	(95% CI)
*Sex*				
Female	Reference		Reference	
Male	0.71	(0.43–1.16)	0.34	(0.14–0.84)
Age	1.01	(0.96–1.05)	1.03	(0.98–1.09)
Height	1	(0.97–1.03)	1.14	(0.91–1.43)
Weight	1	(0.98–1.03)	0.87	(0.65–1.16)
Body mass index, kg/m^2^	1.02	(0.94–1.1)	1.44	(0.71–2.92)
*Current smoking*				
No	Reference		Reference	
Yes	0.51	(0.26–0.99)	0.62	(0.3–1.28)
*Recent health screen*				
No	Reference		Reference	
Yes	2.13	(1.31–3.47)	1.97	(1.15–3.35)
*Spous_yes*				
No	Reference		Reference	
Yes	1.52	(0.92–2.53)	1.94	(0.96–3.93)
*Insurance*				
National health insurance	Reference		Reference	
Medical aid	0.32	(0.15–0.67)	0.34	(0.15–0.79)
*Insurance_private*				
No	Reference		Reference	
Yes	1.12	(0.62–2.04)	1.16	(0.61–2.2)
*Obesity*				
Under BMI 25 kg/m^2^	Reference		Reference	
≥ BMI 25 kg/m^2^	1.07	(0.65–1.75)	1.07	(0.47–2.47)
*Routine exerciser*				
No	Reference		Reference	
Yes	0.8	(0.49–1.3)	0.85	(0.51–1.44)
*Hypertension*				
No	Reference		Reference	
Yes	0.94	(0.54–1.64)	0.83	(0.46–1.51)
*Diabetes mellitus*				
No	Reference		Reference	
Yes	1.03	(0.61–1.75)	1.23	(0.7–2.17)
*Hyperlipidemia*				
No	Reference		Reference	
Yes	1.2	(0.69–2.07)	1.02	(0.56–1.84)
*History of cancer*				
No	Reference		Reference	
Yes	1.69	(0.58–4.9)	2.14	(0.65–7.03)

**Table 4:** Multivariate logistic regression analysis to identify factors associated with influenza vaccination status among Korean adults with cardiovascular disease (< 65 years)

	Crude OR	(95% CI)	Adjusted OR	(95% CI)
*Sex*				
Female	Reference		Reference	
Male	0.47	(0.29–0.77)	0.45	(0.18–1.09)
Age	1.07	(1.03–1.12)	1.06	(1.01–1.12)
Height	0.97	(0.95–1.0)	1	(0.76–1.31)
Weight	1	(0.98–1.03)	1.03	(0.74–1.45)
Body mass index, kg/m^2^	1.08	(1.0–1.18)	1.05	(0.42–2.59)
*Current smoking*				
No	Reference		Reference	
Yes	0.36	(0.19–0.69)	0.51	(0.24–1.1)
*Recent health screen*				
No	Reference		Reference	
Yes	3.37	(1.87–6.07)	3.01	(1.56–5.79)
*Spous_yes*				
No	Reference		Reference	
Yes	0.5	(0.23–1.08)	0.57	(0.24–1.36)
*Insurance*				
National health insurance	Reference		Reference	
Medical aid	0.8	(0.2–3.27)	1.01	(0.19–5.47)
*Insurance_private*				
No	Reference		Reference	
Yes	1.31	(0.78–2.2)	1.53	(0.85–2.76)
*Obesity*				
under BMI 25 kg/m^2^	Reference		Reference	
≥ BMI 25 kg/m^2^	1.25	(0.77–2.03)	0.74	(0.31–1.74)
*Routine exerciser*				
No	Reference		Reference	
Yes	1.16	(0.71–1.89)	1.14	(0.67–1.97)
*Hypertension*				
No	Reference		Reference	
Yes	1.57	(0.97–2.55)	1.22	(0.69–2.15)
*Diabetes mellitus*				
No	Reference		Reference	
Yes	1.55	(0.87–2.76)	1.59	(0.81–3.11)
*Hyperlipidemia*				
No	Reference		Reference	
Yes	1.34	(0.81–2.21)	1.26	(0.7–2.27)
*History of cancer*				
No	Reference		Reference	
Yes	1.08	(0.3–3.91)	1.12	(0.28–4.49)

In CVD patients aged < 65 years, age (OR, 1.06; 95% CI, 1.01–1.12) and recent health screening (OR, 3.01; 95% CI, 1.56–5.79) are the two most significant factors associated with influenza vaccination (Table 4). Older CVD patients who have undergone recent health screening in this group are more likely to show high adherence to influenza vaccination.

**Fig. 2:**
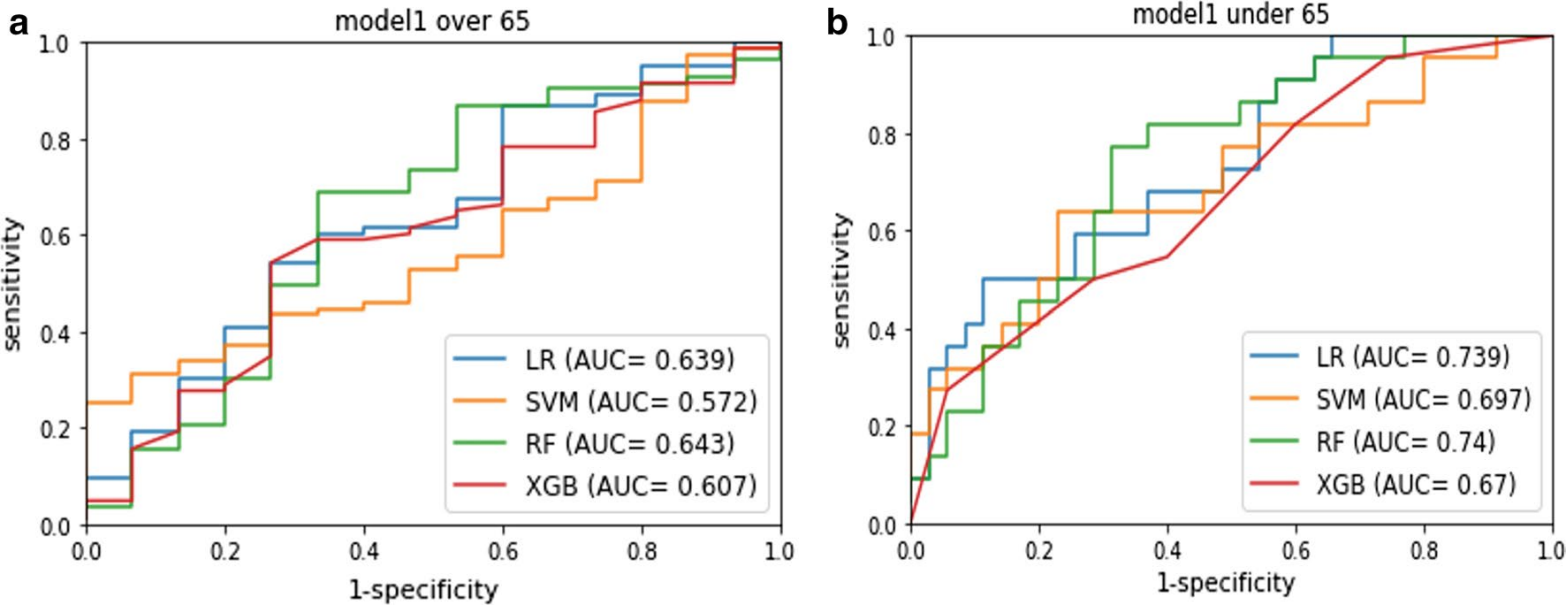
Receiver operation characteristics. Abbreviations: LR, logistic regression; SVM, support vector machine; RF, Random Forest; XGB, extreme gradient boosting; TP, true positive; FN, false negative; FP, false positive; TN, true negative; AUC, area under ROC curve

Table 5 and Fig. 2 show the performance of the LR and the machine learning models in predicting influenza vaccination adherence on the test datasets. The AUC of LR is comparable to that of the three machine learning models. RF shows the best performance, with an AUC of 0.643 for age ≥ 65 years and AUC of 0.740 for age < 65 years; however, the difference from the other models is not significant.

**Table 5:** Confusion matrix for prediction models (Test dataset for the over-65 age group and under 65 age group)

	Korean adult with cardiovascular disease (≥ 65 yrs)					
	TP	FN	FP	TN	Accuracy	AUC
LR	1	14	3	80	0.827	0.639
SVM	2	13	9	74	0.776	0.572
RF	0	15	0	83	0.847	0.643
XGB	0	15	0	83	0.847	0.607

	Korean adult with cardiovascular disease (< 65 yrs)					
	TP	FN	FP	TN	Accuracy	AUC
LR	35	0	21	1	0.632	0.739
SVM	31	4	14	8	0.684	0.697
RF	31	4	16	6	0.649	0.74
XGB	35	0	22	0	0.614	0.67

## Discussion

Since patients with CVD present higher morbidity and mortality when infected by seasonal influenza, it is strongly recommended for CVD patients to receive influenza vaccination. However, the influenza vaccination adherence for this population is not high, especially among the non-elderly [8]. Recently, the use of machine learning has received increasing attention over time, especially in medical science where a tremendous amount of data has been generated from research and clinical practice [10, 11].

Using data from KHANES V, 815 adult CVD patients with accessible data were selected to develop machine learning model to identify low adherence to influenza vaccination. We developed several classification model using different machine learning techniques. With these model, CVD patients at risk of influenza infection can be identified so that health care and the promotion of influenza vaccination can be enhanced for these high-risk patients. Because a well-known factor associated with influenza vaccination is the age [8], since the vaccination is free for adults above the age of 65 in Korea, separate classification models were developed for the two age groups of age ≥ 65 years and age < 65 years.

Among the elderly CVD patients (age ≥ 65 years), the sex and national insurance type significantly affect influenza vaccination adherence. On the other hand, for the < 65 age group, the age and recent health screening status are significant factors. Using socio-demographic variables, health-related lifestyle factors, and health status, prediction models were generated using four machine learning techniques, namely, LR, SVM, RF, and XGB. Using a tenfold cross validation, the dataset was split into ten equally sized random groups; nine groups were used as the training sets for the prediction models, and the remaining group was used as the test dataset, and to train the four prediction models. The diagnostic performance of LR is comparable to that of the three machine learning models, and the RF prediction model shows the best AUC for predicting the vaccination status among CVD patients in both age groups.

From KNHANES III to VI, influenza vaccination rates have steadily increased from 2005 to 2014 in South Korea and high vaccination coverage was associated with female gender, rural residence, low education level, high income, and increasing number of chronic diseases [12]. Factors associated with influenza vaccination coverage was also analyzed among elderly, patients with diabetes, chronic obstructive pulmonary disease, asthma, CVD, cancer survivors using the traditional multivariable logistic regression analysis on KNHANES dataset [8, 13–17]. In the present study, we used machine learning techniques to identify factors associated with low adherence to influenza vaccination among Korean adults with CVD. Machine learning has advantage over traditional statistical methods since it can address a lot of variable information and can easily address complex interactions between these variables.

Influenza vaccination plays an important role in protecting high-risk population, which is a group that is particularly vulnerable for COVID-19. A number of papers that highlight the significant benefits of influenza vaccination in the current COVID-19 pandemic [18]. Influenza vaccination reduced COVID-19 infection risk in COVID-19 infection prediction model [19]. The influenza vaccination would enhance the management of respiratory outbreaks coinciding with the peak flu season, thus enabling more efficient use of healthcare resources [20].

## Limitation

There are several limitations to this study. First, this study has a small sample size. A reliable way to validate the performance of a machine learning model is to train the model with available data and assess its classification performance using newly collected data or a separate dataset. Using unseen data to evaluate a machine learning model gives an unbiased estimate of its performance. We developed machine learning models to classify 16 survey-based variables (most of the variables were binary variables) in a total of 778 adults with CVD using data from KHANES. These 778 adults were subdivided into two groups according to their ages: adults aged ≥ 65 years (n = 496) and adults aged < 65 years (n = 282). Because of the small sample size, the receiver operating characteristics (ROC) curve is not a smooth curve but a step graph. When validation with a separate dataset is not feasible because of the small sample size, K-Fold cross-validation is very economical as it uses all the data for training and also reuses all the data for validation. Cross-validation is a common solution when the available datasets are limited; instead of training a fixed model only once as in the train/test split, several models are iteratively developed using different portions of the data on the cross-validation method. Second, the dataset was imbalanced. Imbalanced data is an unequally distributed dataset in which a certain class of data is significantly larger in quantity than the other data classes [21]. Owing to this disproportionate dataset, the prediction model tends to have good accuracy on the majority class data but poor accuracy on the rest of the data. That is, the prediction model can be inaccurate and biased towards the majority class data. In this study, among the 778 CVD patients, 526 received influenza vaccination and the remaining 252 did not. Because the proportion of people with influenza vaccination is quite different between the two age groups, there is a possibility of learning bias in the machine learning models. Due to these limitations, further studies using larger sample sizes and balanced datasets will be needed.

## Future directions

Influenza vaccination is an important public health goal for protecting high-risk cardiovascular patients in the context of the ongoing COVID-19 pandemic. It is very important to identify the low adherence group and implement strategies to increase influenza vaccination coverage.

## Conclusion

Machine learning models showed comparable performance in identifying low influenza vaccination adherence among CVD patients. Machine learning model might be used to enhance the health care for high-risk CVD patients by identifying patients at risk of influenza infection and promoting influenza vaccination to them.

## Data Availability

The public access to the database is open; the dataset and questionnaire is provided with guidelines for calculating a health-related index through the KCDC online site (https://knhanes.cdc.go.kr/knhanes/eng/index.do).

## Abbreviations

CVD: Cardiovascular disease
KNHANES: Korea National Health and Nutrition Examination Survey
LR: Logistic regression
RF: Random forest
SVM: Support vector machine
XGB: Extreme gradient boosting
WHO: World Health Organization
KCDC: Korea Center for Disease Control and Prevention
BMI: Body mass index
TR: True positive
FN: False negative
FP: False positive
TN: True negative
AUC: Receiver operating characteristic curve
OR: Odds ratio
CI: Confidence interval
ROC: Receiver operating characteristics
